# The Effects of Play Behavior, Feeding, and Time of Day on Salivary Concentrations of sIgA in Calves

**DOI:** 10.3390/ani9090657

**Published:** 2019-09-05

**Authors:** Katrin Spiesberger, Stephanie Lürzel, Martina Patzl, Andreas Futschik, Susanne Waiblinger

**Affiliations:** 1 Institute of Animal Welfare Science, Department for Farm Animals and Veterinary Public Health, University of Veterinary Medicine, Vienna, Veterinärplatz 1, 1210 Vienna, Austria (K.S.) (S.W.); 2 Institute of Immunology, Department for Pathobiology, University of Veterinary Medicine, Vienna, Veterinärplatz 1, 1210 Vienna, Austria; 3 Department of Applied Statistics, JK University Linz, Altenberger Str. 69, 4040 Linz, Austria

**Keywords:** immunoglobulin A, saliva, cattle, emotions, circadian rhythm

## Abstract

**Simple Summary:**

The focus of animal welfare science has shifted over the last decades from efforts to avoid negative states to ways of allowing animals the experience of positive emotions. The emotional state of an animal interacts with its immune system. Secretory immunoglobulin A, a class of antibodies present on mucosal surfaces and acting as the first line of defense against infections, is influenced by positive and negative emotions in humans; the few studies of its association with emotions in animals focused almost exclusively on the impact of negative emotions and yielded conflicting results. We present the first study that focuses on salivary immunoglobulin A to investigate a possible relationship between positive emotions and immune functioning in calves. We detected a circadian rhythm of immunoglobulin A concentrations, with lowest levels at 14:00 h. Immunoglobulin A concentrations were decreased directly after feeding, possibly due to increased saliva flow rates, and we did not find higher immunoglobulin A concentrations after play. The results are important for the design of future studies of positive emotions, although they do not support immunoglobulin A as an indicator of positive emotional states.

**Abstract:**

The focus of animal welfare science has shifted over the last decades from efforts to avoid negative states to ways of allowing animals the experience of positive emotions. They may influence physiological processes in farmed animals, potentially providing health benefits; in addition, the physiological changes might be used as indicators of emotional states. We investigated calves’ salivary secretory immunoglobulin A (sIgA) concentrations with regard to a possible circadian rhythm and two situations that elicit positive emotions. Ten saliva samples of 14 calves were taken on two consecutive days; within the course of a day we observed a significant decline in salivary sIgA concentrations at 14:00 h. Further, we probed the animals before and after milk feeding and, contrarily to our prediction, detected lower sIgA concentrations 5 min after feeding than 15 min before. A probable explanation might be an increase in salivary flow rate caused by milk ingestion. We also took samples before and after we stimulated play behavior in calves. There was no significant difference in sIgA concentrations between samples taken before and after play. Although there was a significant correlation between the change in sIgA concentrations and the amount of play behavior shown, the correlation depended on an unexpected decrease of sIgA in animals that played little, and thus, does not support our hypothesis. In general, the data showed a large variability that might arise from different factors that are difficult to standardize in animals. Thus, the use of salivary sIgA concentrations as a marker of positive emotions in calves is not supported conclusively by the present data.

## 1. Introduction

Although the focus of animal welfare science has traditionally been mainly on negative aspects, it recently has shifted to include the assessment of positive welfare and thus, positive emotions [1]. In animal emotion research, the actual interest lies in the conscious subjective experience, characterized by arousal and valence [1,2]. Although this subjective component is not directly accessible to science, the corresponding behavioral, physiological, and cognitive components can be measured, making it possible to assess affective states in animals (e.g., [3]).

The influence of stress and affective states on immune functioning is well known, mostly in humans [4,5] but also in animals [6,7]. Measures of immune functioning have been proposed for assessment of affective states in animals [1]. One of the numerous indicators of immune functioning is immunoglobulin A (IgA) [8]. Secretory immunoglobulin A (sIgA) is present on most mucosal surfaces, providing the first line of defense of the organism against infective agents like bacteria and viruses [8]. In animal welfare studies, it has mainly been measured in saliva (e.g., [9]) and feces (e.g., [10]), but also in milk [11]. Salivary sIgA concentrations were shown to react within 10–15 min after eliciting an emotional state in animals [9,12].

In general, data on sIgA concentrations after the experience of either positive or negative emotions in animals are limited (for a review, see [8]). The few published studies focused mostly on negative emotions, with conflicting results. In dogs, decreased sIgA concentrations were reported after stressful situations [9,13]; the effect appears to be age-dependent, since sIgA concentrations were increased in puppies after stress [14]. In male rats, social housing conditions with different levels of competition and mating opportunities influenced salivary sIgA concentrations, with conditions deemed more favorable (presence of a female and bedding) leading to a steep decrease and subsequently to a gradual increase and less favorable conditions (group-housing with other males) to a decrease [15]. In pigs, an increase in salivary sIgA was detected after restraint stress [12]. Regarding cattle, there is one study that examined the effect of removal of conspecifics on sIgA [11], which is a stressful event according to behavioral observations. After the removal, they found no difference in milk sIgA concentrations between cows that were associated with the removed animals or not; there was an increase in serum IgA concentrations in calves and young bulls after removal of their pen mates, but sample sizes were very small and there were no control groups [11].

Although there are strong indications for an increase of salivary sIgA in response to positive emotions in humans (e.g., [16,17]), there are few studies in animals. Regarding a potential effect of positive emotions, sIgA concentrations in the feces of sheltered cats that had been stroked or whose behaviors were classified as positive were elevated [10,18]. To be able to interpret results on sIgA correctly, the circadian rhythm should be considered. The circadian rhythm may vary strongly between species [8] and although there are studies in dogs [9] and pigs [12], no data are available for cattle to date. The first aim of our study was therefore to investigate a potential circadian rhythm of salivary sIgA dynamics in calves. The second and main aim was to elucidate the effect of positively valenced emotions on salivary sIgA in calves. We hypothesized an increase of sIgA during milk feeding as well as during experimentally induced play behavior, which are both situations that are associated with positive emotions [1].

## 2. Materials and Methods

### 2.1. Subjects, Housing and Management

Twelve Austrian Simmental (eight females, four males) and two Holstein calves (both female) were tested for changes in salivary sIgA concentrations during play at an average age of 61 ± 9 days (mean ± SD). From 14 days of age, the calves were housed together in a deep litter barn at the Teaching and Research Estate Kremesberg of the Vetmeduni Vienna (Pottenstein, Austria). They were kept in three groups of six to eight animals together with calves that were not involved in the study. We tested two animals out of six in group A, eight animals out of eight in group B and four out of seven in group C. Each group was housed in a 7 × 5 m deep litter pen, including a 12.5 m^2^ area of 1.3 m height that was separated by a transparent strip curtain, and eight individual feeding stalls (0.5 × 1.7 m^2^) with wooden walls and concrete floor. The calves were fed with pasteurized milk twice a day, around 07:30 h and 18:00 h, with 3–5 L of milk per feeding, depending on age. During milk feeding until approximately 30 min after feeding, the calves were confined in the feeding stalls by gates to reduce allosucking; during the rest of the day, they could enter and leave the stalls freely. Water, calf feed (Kälber Start Vital; Garant, Pöchlarn, Austria) and hay were provided ad libitum. All calves were disbudded with a hot iron by a veterinarian at an age of 4–5 weeks. They were sedated (Sedaxylan, 20 mg Xylazin/mL: 0.1 mg Xylazin/kg body weight) and received local anesthesia (Procamidor 2% Procain: each side ca. 5 mL) and analgesia (Rifen, 100 mg Ketoprofen/mL: 3 mg Ketoprofen/kg body weight). Disbudding was performed at least 14 days before the habituation period started. The study was discussed and approved by the institutional ethics committee in accordance with Good Scientific Practice (GSP) guidelines [19] and national legislation (project number 01/03/97/2014).

### 2.2. Experimental Procedures

**Figure 1:**
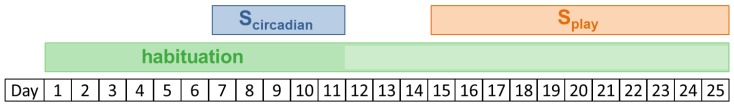
Schematic view of experimental phases. After the first days of habituation, saliva was sampled for detecting a circadian rhythm (S_circadian_) on two consecutive days for each calf (between day 7 and day 11). Saliva sampling before and after play (S_play_) took place on days 15 to 25. During the first 11 days, the habituation procedures were performed daily (dark green). To maintain familiarity of the calves with the experimenters, habituation was continued after S_circadian_ on every second day (light green).

The experiment took place between March and May 2014 (Figure 1). After the habituation period, the salivary sIgA concentrations of the subjects were determined over the course of the day on two consecutive days per calf in order to determine the circadian sIgA rhythm and the possible influence of milk feeding. Between 4 and 14 days later (depending on temporal constraints due to farm procedures), saliva samples were taken before and after induced play and behavior was directly observed as well as video-recorded for later, detailed analysis.

Calves were habituated to human contact and the procedure of saliva sampling by one of two female experimenters (170 cm, blonde, and 176 cm, brown hair and glasses, both dressed in dark green overalls) for 1–2 h twice a day for 11 consecutive days (Figure 1). First, the experimenter entered the box and initially waited for the calves to seek contact. As soon as the animals approached the experimenter voluntarily and did not show overt avoidance reactions, she also initiated body contact, stroked them, and allowed them to suck her fingers. If the calf pulled away after initial contact, the experimenter waited for the calf to approach again. Furthermore, the procedure of saliva sampling was simulated by placing the sampling device shortly into the calves’ mouths. The experimenters did not encourage the subjects to play nor actively played with them during the habituation period. Calves were considered as sufficiently habituated when they approached the experimenter readily and/or let themselves be touched. After taking the samples for the analysis of the circadian rhythm, the procedure of habituation was continued every second day for 14 more days (day 11 to day 25) until all the samples in the context of the play situation were taken (Figure 1).

**Figure 2:**
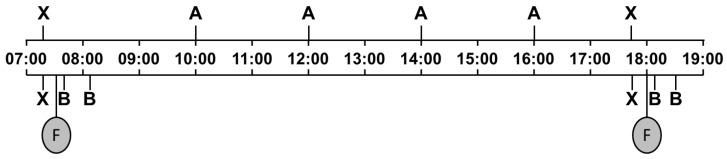
Schedule for saliva sampling in calves. F, morning and evening milk feeding (07:30 h and 18:00 h); A, saliva sampling to determine circadian rhythm at fixed times of day; B, saliva sampling to determine effect of feeding, 5 and 30 min after the end of milk feeding; X, saliva sampling serving both purposes.

On two consecutive days, saliva samples were taken to gain general information about each calf’s salivary sIgA concentration during the course of a day and the influence of feeding. Depending on the calves’ state of habituation, they were sampled after 7–11 days of habituation, and on any given day, two to four calves were sampled. Six samples were taken over the course of each day (Figure 2), at 07:15 h (before morning milk feeding), 10:00 h, 12:00 h, 14:00 h, 16:00 h, and 17:45 h (before evening milk feeding). The samples at 07:15 h and 17:45 h were also used as a baseline in the analysis of a possible effect of feeding on sIgA concentrations. Additional samples were taken 5 and 30 min after the end of milk feeding in the morning and in the evening, resulting in a total of 280 saliva samples. The removal of the feeding bucket as soon as it was empty marked the end of milk feeding, which varied among the tested calves, depending on the amount of milk fed and each calf’s drinking behavior. As a circadian rhythm with a significant change within 35 min is highly improbable, we refrained from including a control condition for feeding; a “no feeding” condition would not have been not valid control, as the animals would have anticipated to be fed at this time of day, and thus, there might have been effects of emotional state (frustration), saliva flow and possibly other factors on sIgA concentrations.

Play tests were performed 4 to 14 days later, twice a day, around 10:15 h as well as 12:30 h, i.e., late enough to exclude a possible influence of feeding. Every calf was tested maximally two times per day and in up to four tests in total. Play behavior cannot be triggered reliably: Sometimes some calves are not motivated to play, whereas at other times, several calves will play at the same time. In addition, play behavior is often contagious [20], i.e., if one calf starts playing, others will join in [21,22]. Thus, we aimed to exploit the effects of emotional contagion or social facilitation and tested several calves in the same pen at the same time. Usually two to four calves were tested per play session depending on the number of experimental animals kept in the pen. For the test, an experimenter entered the box and immediately took a baseline saliva sample of the subjects to be tested. Then locomotor play behavior was encouraged by the experimenter moving between the calves. The way of moving included running and jumping with relatively slow, exaggerated movements, sometimes but not always including eye contact with the calves. Further, the experimenter initiated physical contact with the calves to induce play fighting behavior: She touched or rubbed a calf’s forehead and progressed to pushing if the animal started to perform head pushing or rubbing. In the meanwhile, the other experimenter manually recorded the frequency of play behavior shown by the subjects to be tested. Depending on whether and when such behavior was shown—according to the second experimenter’s observations—a second saliva sample was taken 15–45 min after the baseline sample; the experimenter aimed to take it within 8 min after play behavior was shown [23].

Saliva was sampled using Salivettes^®^ (Sarstedt; Nürnbrecht, Germany). A rubber teat from a bucket was put over dressing forceps so that approximately 2 cm of the forceps were visible outside of the rubber teat. A cotton swab was then gripped with the forceps. For taking a saliva sample, the experimenter approached a calf in its home pen and carefully placed the forceps into its mouth for at least 30 s, if possible without restraining the subject. Most often, simultaneous stroking of the calf was sufficient to make it tolerate the sampling procedure; only for 29 out of 334 samples, the subject had to be restrained by the experimenter. If restraint was necessary, the calf was either confined in a feeding stall or held by the experimenter by putting one arm around its neck. All samples that were used to analyze the effect of feeding on IgA were taken while the animals were in the feeding stalls. Samples were immediately put on ice and frozen at −20 °C within a maximum of 15 min.

### 2.3. Analysis of sIgA Concentrations

Saliva samples were thawed and centrifuged for 5 min (1000× *g*, 4 °C). Supernatant was taken and samples were diluted in Tween-TRIS buffer (50 mM Tris, 0.14 M NaCl, 0.05% Tween 20, pH 8) at ratios of 1:1000, 1:2500, 1:5000, and 1:10,000. Salivary sIgA concentrations were determined using the Bovine IgA ELISA Quantitation Set according to the manufacturer’s protocol (Bethyl Laboratories; Montgomery, AL, USA). Standard and sample dilutions were analyzed in duplicates. Optical densities (OD) at 450 nm were measured using an ELISA reader (Epoch Microplate Spectrophotometer, Biotek; Bad Friedrichshall, Germany). The OD values and log IgA concentrations were plotted using a four-parameter logistic regression model analysis.

### 2.4. Observation of Play Behavior

Behavior was observed directly and coded from video recordings after training by one of the experimenters, using an ethogram (Table 1) based on previous descriptions [21,22,24]. It included social and locomotor play behavior patterns as well as avoidance, because the occurrence of repeated avoidance may indicate a certain level of fear that could influence salivary sIgA concentrations. Head rubbing towards objects in the environment has been described in the context of play behavior [24]. As we did not expect the experimenter to induce object play in the calves, we focused on social play and included social head rubbing because it is often shown in the context of play behavior and there are gradual transitions between head rubbing and head pushing. In addition to this playful component, it is also an affiliative behavior [22] and thus highly likely to contribute to or indicate a positive affective state [1], which we intended to induce. Direct observations were necessary to determine when the second salivary sample should be taken, whereas video observations allowed coding play and avoidance behavior in detail. Behavior was observed directly by an experimenter sitting in front of the tested calves’ pen at about 2 m height in the feeding alley. For video recordings, two cameras (EcoLine TV7204, Abus; Wiener Neudorf, Austria) were placed at 3 m height, one at the left and one at the right corner of the front side of the pens. The observation started as soon as the experimenter entered the pen and ended when she left it. Only the behavior that was shown between the two saliva samples was analyzed. Behavior was coded using the Interact^®^ software (V14.0, Mangold; Arnstorf, Germany), recording frequencies and durations of behavioral patterns (Table 1). The observer also recorded when saliva samples were taken. Both types of behavioral observations were done using behavior sampling and continuous recording [25].

### 2.5. Statistical Analysis

All statistical analyses were performed and graphs were created using the R statistical environment, versions 3.2.3 and 3.4.3 [26]. In all boxplots, the length of the box refers to the interquartile range (IQR) and the horizontal line represents the median value. The end of the lower whisker represents the lowest data point still within 1.5 × IQR from the lower quartile and the end of the higher whisker represents the highest data point still within the 1.5 × IQR from the upper quartile. Values outside this range are considered outliers and depicted as open dots.

The experimental unit was the individual calf. To analyze the data gained from saliva samples taken over the course of the day (2 × 10 samples per calf), linear mixed models (LMM) were calculated. In all models, the animal nested in the group was included as a random effect. For the analysis of changes in circadian sIgA concentrations, time of sampling, sex, day of sampling and their interactions were included as fixed effects in the full model. After model selection using the Akaike Information Criterion (AIC), only time was included as a fixed effect. For the analysis of a possible influence of feeding on IgA concentrations, sample number (SB, baseline sample taken before feeding; S5, taken 5 min after feeding; S30, taken 30 min after feeding), time of day (AM; PM), sex, day of sampling and their interactions were included as fixed effects in the full model. After model selection using the AIC, only sample number was included as a fixed effect. Normal distribution of residuals was checked by means of a Q-Q plot, the homogeneity of variance of the residuals by plotting the residuals against the predicted values resulting from the model. sIgA values were log-transformed to fulfill the model assumptions. If there were outliers in the residuals (>3 SD), the model was calculated again without the corresponding observations in order to determine whether they have a strong influence on the results. This was never the case and the models calculated before the exclusion of outliers are presented. Post-hoc comparisons were calculated with Tukey adjustment.

One calf was excluded from analysis of play behavior and its effect on sIgA due to loss of video data. To summarize play behavior, a duration of 1 s was assigned to each occurrence of behaviors recorded as events, because their duration would usually be around 1 s as observed during the habituation phase. A play score was then calculated for each play test of each calf by adding the durations of all play behaviors, divided by the time (in min) between the two saliva samples to account for different durations of observation. If several play tests were conducted with a calf, only the test with the highest play score was chosen for the analysis of salivary sIgA concentrations. If a calf was tested only once, the samples were included in the analysis regardless of the calf’s play score. A one-sample sign test was performed to compare salivary sIgA concentrations before and after playing. Since the average duration of a play test was relatively short (mean 24.9 ± SD 7.5 min), sIgA concentrations were not expected to be influenced by the circadian rhythm, and we refrained from controlling for an effect of time. Delta sIgA was calculated (ΔsIgA = sIgA concentrations determined after the play test minus baseline sIgA concentrations determined before the play test) and a possible correlation of the play score and ΔsIgA was investigated using Spearman’s rank correlation test, as it is insensitive against outliers and we did not expect a strictly linear correlation. The same test was used for calculating the correlation between play score and avoidance behavior.

## 3. Results

### 3.1. Effect of Time of Day

**Figure 3:**
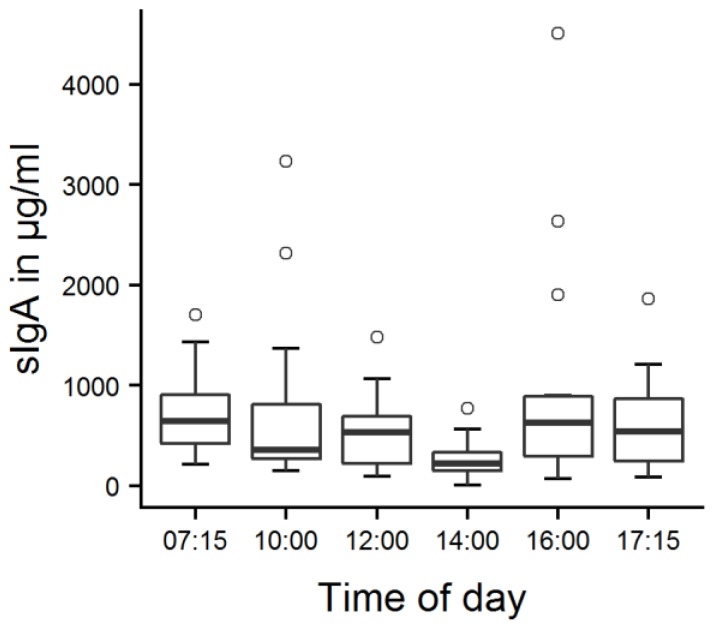
Calves’ salivary sIgA concentrations at different points in time. Fourteen calves were tested on two consecutive days; values were averaged per calf and point in time. There was a main effect of time (LMM, *p* < 0.001); the values at 14:00 were significantly lower than the values at all other points in time according to Tukey contrasts (*p* = 0.001–0.022).

The mean salivary sIgA concentration was 689 ± 1115 µg/mL (mean ± SD) on the first day and 636 ± 803 µg/mL on the second day. Three outliers with sIgA concentrations exceeding 3000 µg/mL were observed on the first day and one outlier on the second day (Table S1). There was a significant effect of time (Figure 3, LMM, F_5149_ = 5.4, *p* < 0.001), with lower sIgA concentrations at 14:00 h than at all other points in time (*p* = 0.001–0.022).

### 3.2. Effect of Feeding

**Figure 4:**
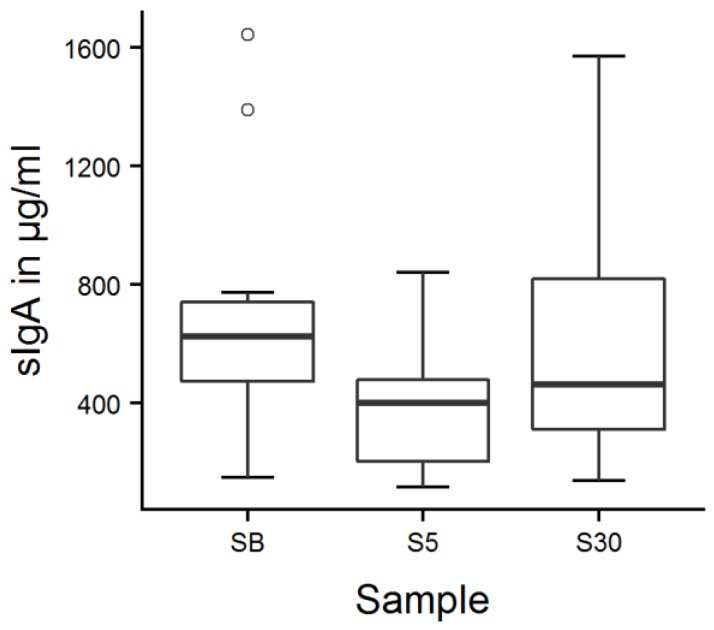
Calves’ salivary sIgA concentrations around feeding time. Fourteen calves were sampled 15 min before (SB), 5 min (S5), as well as 30 min after the milk feeding (S30), which took place twice a day on two consecutive days; values were averaged per calf and point in time. There was a main effect of sample number (LMM, *p* = 0.007); values decreased from SB to S5 (*p* = 0.007) and increased from S5 to S30 (*p* = 0.049) according to Tukey contrasts.

The mean sIgA concentration was 533 ± 504 µg/mL on the first day and 555 ± 531 µg/mL on the second day. There was a main effect of sample number on salivary sIgA concentrations (Figure 4, LMM, F_2152_ = 5.2, *p* = 0.007): salivary sIgA concentrations were lowest 5 min after milk feeding (Table S2; *p* = 0.007) and increased afterwards (*p* = 0.049).

### 3.3. Play Behavior and Its Effect on sIgA Concentration

**Figure 5:**
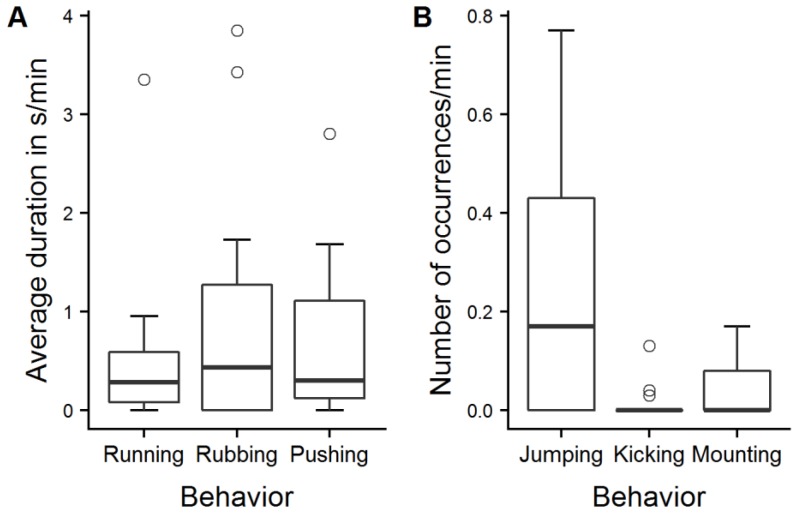
Play behavior. Average duration in s/min (**A**) and number of occurrences/min (**B**) of different patterns of play behavior displayed by 13 calves in the play test selected for evaluation of the effect of play behavior on sIgA.

Within the play test, it took the experimenter on average 3.0 ± 2.5 min to take the baseline saliva sample after she entered the pen. Out of 13 calves, two showed short occurrences (<30 s) of play behavior in the time from entering the pen until the experimenter took the sample. On average, the calves showed their first play behavior 5.8 ± 2.9 min after the baseline saliva sample was taken. The second saliva sample was taken 24.9 ± 7.5 min after the baseline sample. Between the two samplings, the experimenter induced play behavior (Figure 5; Table S3). On average, 18.9 ± 7.9 min elapsed between the calves’ first play behavior and the collection of the second saliva sample and 3.1 ± 3.3 min between the last play behavior and the second sample.

**Figure 6:**
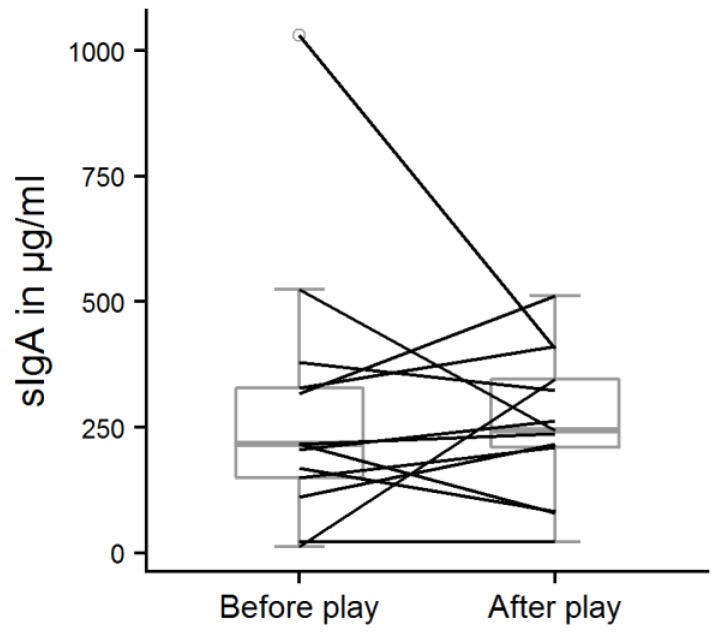
Salivary sIgA concentrations before and after the play test situation. One-sample sign test, n = 13, s = 7, *p* = 0.39.

**Figure 7:**
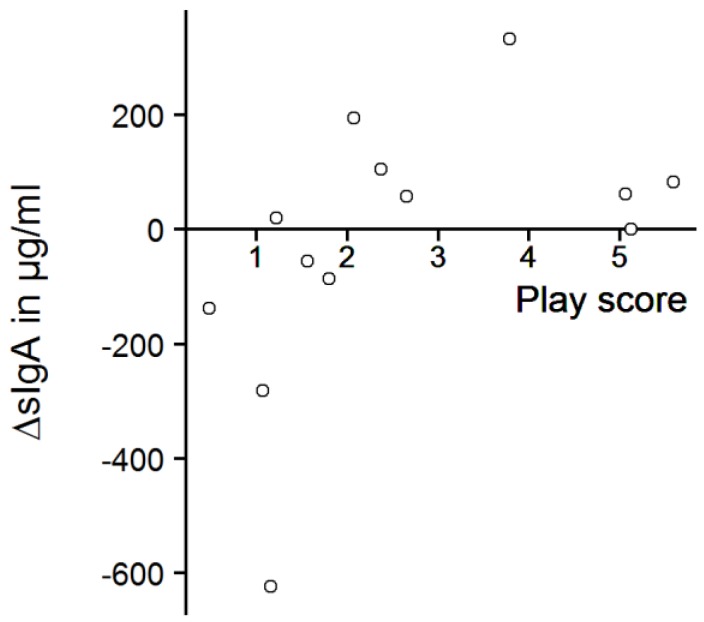
ΔsIgA and play score. ΔsIgA = (sIgA concentration after play test) minus (sIgA concentration before play test); Spearman’s rank correlation, n = 13, r = 0.69, *p* = 0.012.

The play score ranged from 0.5 to 5.6, with a mean score of 2.6 ± 1.7. In total, avoidance behavior was recorded 37 times (0.14 ± 0.18 times/min), but it was mostly shown by only two calves (play scores: 1.6 and 3.8). The calves that played most (play scores of 5.6 and 5.1) never avoided the experimenter in the play test. However, there was no significant correlation between avoidance behavior and play score (Spearman’s rank correlation, r = −0.28, *p* = 0.35). The salivary sIgA concentrations of the calves probed before the play test ranged from 13 to 1030 µg/mL (mean: 283 ± 265 µg/mL). After the play test, sIgA concentrations were between 22 and 512 µg/mL (mean: 258 ± 143 µg/mL). There was no significant difference between sIgA concentrations before and after the play test (Figure 6, one-sample sign test, s = 7, *p* = 0.39) but we found a strong, significant correlation between the play score and ΔsIgA (Figure 7, Spearman’s rank correlation, r = 0.69, *p* = 0.012).

## 4. Discussion

### 4.1. Effect of Time of Day

There was an effect of time on calves’ salivary sIgA concentrations, with the lowest concentrations detected at 14:00 h, compared to all other times; sIgA seems thus to undergo circadian changes. In humans, salivary sIgA was shown to decline steadily during the course of the day [27], but little is known about animals’ circadian changes of sIgA concentrations [8]. Contrary to our findings, studies in pigs and dogs demonstrated lower salivary sIgA in the morning and elevated salivary IgA concentrations over the midday hours, which declined again in the late afternoon [9,12]; rats housed in metabolic cages excreted the highest amounts of fecal sIgA between 20:00 h and 02:00 h, which corresponds to a major part of their active phase [28]. The different results might be due to general differences between species or their different diurnal rhythms of activity or arousal. In calves, rumination might also influence salivary sIgA concentrations, since it may result in enhanced saliva flow rates that lead to a decline in sIgA concentration [29], and additionally in mixing of rumen content with saliva.

In general, the data showed a high variability between and within the calves as well as large outliers. From a technical point of view, the large variability of our data could be influenced by the restricted possibilities of standardization of the sampling procedures in animals. In humans, saliva sampling is a well-established procedure that includes a standardized behavioral protocol, resulting in saliva collected over a defined time span and under comparable conditions [30]. Saliva sampling is far more difficult to standardize in animals because they cannot be instructed to behave in the way a human can be. Consequently, the time span of sampling differs, as some animals interrupt the sampling. Also, oral behavior before the sampling procedure could not be controlled in the present study, which is important since salivary sIgA is dependent on the saliva flow rate, which is influenced by mechanical, gustatory and olfactory factors [31].

Salivary sIgA concentrations can change in response to positive or negative emotional events and salivary IgA concentrations react to emotional stimulation already several minutes after the onset of stimulation [9,32]. Since we cannot exclude that tested calves experienced positive or negative emotions before samples were taken, emotional experiences constitute a potential confounder. In future studies, behavior should be observed for several hours before sampling to detect such situations and consider them in data analysis. However, it is unlikely that calves experience strong emotions at consistent times across days, outside of the context of milk feeding.

### 4.2. Effect of Feeding

The examination of salivary sIgA concentrations revealed decreased concentrations directly after feeding, regardless of the tested day, time of feeding or sex of calves. There was no significant difference between the samples taken before and 30 min after the milk feeding. As we rated feeding as a positively valenced event, we would have expected an increase in salivary sIgA concentrations directly after feeding, which was clearly not the case. Food intake stimulates saliva production [31], and with increasing saliva flow rates, salivary sIgA concentration decreased in humans [29]. Thus we suppose that the observed decline in sIgA concentrations resulted from an increase of saliva flow rate during feeding. As it was not possible to determine saliva flow rate in our experiment, there are no data to support this explanation at the moment. Emotional arousal is a component of emotional states that can act as a confounder. As we had no possibility to control for potential differences in arousal in the present experiment, the observed sIgA decline might theoretically also be explained by a state of low arousal caused by post-consummatory satisfaction [2].

Another factor that could have influenced the obtained results is the temporal dynamics of sIgA in saliva. Little information is available about temporal aspects of salivary sIgA, but concentrations were significantly different to baseline at 10 and 15 min after the onset of an emotional stimulus in dogs and pigs, respectively [9,12]. These samples were, however, the first samples taken after the onset of the stimulus, which means that sIgA might react even faster. Pre-stimulus levels were reached 10 min (pigs) and 30 min (dogs) after the end of the stimulus, although still being significantly different from controls in dogs. Again, this was the first recovery sample, so pre-stimulus levels might be attained even faster. As we took the first post-stimulus saliva sample approximately 10 min after the onset of feeding and 5 min after the bucket had been removed, it seems reasonable to assume that we indeed detected the sIgA response to feeding. Nevertheless, there might be differences between species, thus further research is necessary to determine the time course of the salivary sIgA reaction in cattle.

In a follow-up study, a possible sampling effect on our data should be considered; without having a non-feeding control it is not possible to distinguish the feeding effect from a potential sampling effect. Although the calves were habituated to all experimenters and the sampling procedure, we cannot exclude that sampling could have caused a negative affective state or stress in some animals that influenced the results, as discussed in detail in Section 4.3.

### 4.3. Effect of Play Behavior

We did not find any significant differences in calves’ salivary sIgA concentrations between the samples taken before and after the play test. We had assumed that every playing calf experienced positive emotions with a moderate to high level of arousal and predicted an increase in sIgA concentrations, but sIgA concentrations were not generally increased after play behavior was performed, as it increased numerically only in seven of the thirteen tested calves. There was a correlation between play score and change in salivary sIgA concentrations, but the result should be interpreted with caution: the majority of the calves that had played a lot showed numerically higher sIgA concentrations, but the calves that played most showed no or weak elevation of sIgA concentrations. In addition, some of the calves that had shown only little play behavior actually had numerically lower sIgA concentrations at the second sample point, especially the ones with very high starting values. The correlation depends thus strongly on the unexpected decreases, and therefore does not support our hypothesis of increased sIgA values after a positive emotional state.

A possible explanation for the numerical decrease might be that the sampling of saliva caused a negative affective state or stress, which might have decreased sIgA concentrations directly or influenced it via an effect on saliva production. Conversely, this potential negative affective state might have masked the effect of play behavior on sIgA concentrations: in animals that showed little play behavior, the effect of play behavior might not have been strong enough to make up for the initial decrease. It is also possible that the animals that played a lot did not experience negative affect or stress during the sampling, which in turn might have facilitated the stimulation of play behavior. As we did not expect negative effects of the saliva sampling because the animals were usually not restrained during the procedure, there was no situation without play that could have been used to control for the effect of sampling itself. These possible explanations remain thus speculative until a follow-up study will be conducted, including a control for the effect of sampling.

In addition, the relationship between sIgA concentrations and play behavior might be complex. For instance, physical activity could have an influence on salivary sIgA: increased [33] as well as decreased [34] salivary IgA concentrations in humans were found during a period of intense exercise, but to our knowledge, no data on the influence of physical activity on sIgA in animals are available. In the present study, it was not possible to separate the influence of emotions from the influence of physical activity, as play behavior is inextricably linked with physical activity, and most physical activity occurred in the context of play behavior. Another confounding effect might have been anticipation, which might have increased basal sIgA concentration. An indication for anticipation was the behavior of the two calves that started to play when the experimenter entered the pen, even before she started to induce play behavior in the calves. However, it speaks against a confounding effect of anticipation that the calves with the highest basal sIgA concentrations played relatively little (play scores 1.16 and 1.07); if their sIgA concentrations were that high because of a premature rise due to anticipation, we would have expected them to show high levels of play behavior in the test.

Play behavior is not easy to define and quantify in many animal species, as it combines behaviors from different behavioral categories. One might thus argue that the behaviors we recorded in this study were possibly caused by fear of the calves towards humans. However, play behavior in calves encompasses quite specific behaviors, which have been described in detail [21,22,24]. The animals did not show defense behavior, which would have been expressed as aggressive behavior towards the experimenter and might have led to injury. Regarding fear, it is more difficult to distinguish, as running may be caused by fear or occur in the context of play behavior. We took a conservative approach and recorded every movement away from the experimenter that did not include specific behavioral elements clearly indicating play (such as kicking, head shaking) as “avoidance”, if it occurred as a direct reaction to her approach. We can thus be quite sure that we recorded all avoidance events as such and rather under- than overestimated the occurrence of play behavior. Another aspect of this issue is that humans are very adept at picking up subtle, qualitative cues in an animal’s behavior that allow them to draw conclusions about the animal’s emotional state [35,36]; we are confident that we would have recognized fearful behavior.

Experiments involving human contact are always influenced by the animal–human relationship (AHR) [37,38]. A positive AHR was essential for conducting this study, as the experimenters depended on the calves’ cooperation during saliva sampling and play behavior occurs usually only in a relaxed situation [20]. The play experiments were performed by two female experimenters, who were familiar to the animals. Nevertheless the experimenters’ behaviors, which triggered play behavior in some calves, might have induced fear in others. Negative feelings towards the experimenter or the play situation should have been reflected by increased avoidance behavior and the absence of play behavior [20,38]. Although we cannot completely rule out that play behavior might have served as a coping mechanism [39,40], in this case to cope with potential fear towards the experimenter, we consider it highly unlikely in the present study: Although Ahloy-Dallaire et al. [39] present several counter-examples to the wide-spread view that play is associated with positive affect, the type of play behavior shown in the described situations seems to be suitable to actually improve the situation and provide a welfare or fitness benefit, such as social play in socially stressed primates or object-play in nutritionally deprived kittens. In contrast, calves’ primary response towards a truly threatening stimulus should be flight and not play, if we think in terms of evolutionary adaptiveness. Avoidance behavior occurred several times; it was mostly shown by only two calves with low and intermediate play scores. On the other hand, some calves were apparently waiting for the experimenter to enter the pen, immediately seeking body contact and sometimes initiating play behavior, showing no or minimal avoidance behavior. The AHR may have differed in our experimental animals, affecting their emotional state during the interactions and thus possibly their sIgA concentrations.

When it comes to emotions and sIgA, the available literature presents rather conflicting findings. Most of the experiments published on the connection between salivary sIgA and emotions were conducted in humans. There are several studies that indicate that positive emotions lead to increased salivary sIgA concentrations, e.g., [16,17], whereas stress as experienced during a period of academic exams down-regulated sIgA secretion [41]. However, Benham et al. [23] showed increased sIgA concentrations after stress-inducing as well as stress-reducing tasks; it is likely that the intensity and duration of the stressor play a role. Studies in animals neither agree on whether sIgA increases or decreases after positively experienced emotions. Authors reported decreased sIgA concentrations after stress-related situations in adult dogs and rats [9,13,14,15]. In contrast, increased salivary IgA concentrations after stressful situations were obtained in pigs and puppies [12,14]. The influence of positive emotions on salivary sIgA was only described in a small study of cattle: A trend towards increased salivary sIgA concentrations was observed after cows were moved to pasture after a long period of loose housing [42]. As in our study, a high variability in sIgA concentrations was reported, suggested to arise from environmental factors, and an unexpected, slight decrease was found mainly in animals with higher starting values.

The reported diversity between and within the species in salivary sIgA responses to differentially valenced emotions raises the question of the role of arousal in sIgA secretion. According to Mendl et al. [2], situations described as acute stress can be considered as experiences associated with high arousal whereas chronic stress, due to a habitation effect, might be connected to low arousal. To our knowledge, only Guhad and Hau [15] specifically investigated chronic stress in rats and observed decreased sIgA concentrations. All other studies dealt with experimental situations causing acute stress and described salivary IgA increases as well as decreases in highly aroused animals [9,12,14].

## 5. Conclusions

In this study, we detected a circadian rhythm of salivary sIgA concentrations in calves, with lowest concentrations at 14:00 h as well as reduced sIgA concentrations directly after feeding. There was no consistent response in sIgA after play, with both increasing and decreasing concentrations. The large variability of the data and the conflicting results in other mammalian species suggest additional influencing factors such as the sampling procedure, salivary flow rates, affective arousal or the AHR, as well as the current infectious pressure. Our results thus do not support the use of salivary sIgA concentrations as a marker of positive emotions in calves.

## Figures and Tables

**Table 1:** Ethogram of observed behaviors. For running, head pushing and head rubbing, durations were recorded; all other behaviors were recorded as frequencies. Head shaking is a supplemental definition (to distinguish avoidance from playful running that is directed away from the experimenter) and was not recorded on its own, as it usually occurs together with running or jumping. All behaviors except for avoidance were considered play behaviors (described in [21,22,24]).

Behavior	Definition
Running	A calf moves forward faster than walking (trot or gallop).
Jumping	During running: All four legs leave the ground, accompanied by a clear upward movement of the calf. On the highest point of movement, the animal can kick with one or both hind limbs. During standing/walking: In an upward movement, both forelimbs leave the ground and the calf lands with both forelimbs simultaneously. The hind limbs can also move.
Kicking	While standing or walking, the calf kicks with one hind limb.
Mounting	A calf jumps with both forelimbs and lays the front part of its body on the body of another animal or the experimenter. Mounting is also recorded if the attempt is not successful, i.e., the upper body part does not come to rest on the other animal or the experimenter.
Head pushing	A calf puts its forehead against the forehead or head/neck region of another calf or against a body part of the experimenter and pushes. This behavior can also be started with another part of the head than the forehead.
Head rubbing	A calf puts any part of its head, usually the side of the face, against a body part of another calf and rubs it in an up-and-down movement.
Head shaking	Up-and-down or rotational head movements, often in combination and around more than one axis; the movements have no clear direction, e.g., towards flies.
Avoidance	A calf moves away from the experimenter after the experimenter moved towards the calf. This behavior is only recorded if the movement is obviously triggered by the experimenter’s approach. If this experimenter-triggered, averted movement leads to a clearly playful behavior (jumping, kicking, head shaking), it is not recorded as avoidance but as the according play behavior (in case of a quick movement in combination with head shaking, the behavior is recorded as running).

## Supplementary Materials

The following are available online at https://www.mdpi.com/2076-2615/9/9/657/s1, Table S1: IgA concentrations over the course of the day, Table S2: IgA concentrations before and after feeding, Table S3: IgA concentrations and play behavior during the play tests.
